# First person – Nakul Wewhare

**DOI:** 10.1242/bio.061701

**Published:** 2024-10-21

## Abstract

First Person is a series of interviews with the first authors of a selection of papers published in Biology Open, helping researchers promote themselves alongside their papers. Nakul Wewhare is first author on ‘ Individual-specific associations between warble song notes and body movements in budgerigar courtship displays’, published in BiO. Nakul is a BS-MS student in the lab of Dr Anand Krishnan at the Indian Institute of Science Education and Research (IISER), Pune, India, investigating the structure and function of complex vocal sequences in animals.


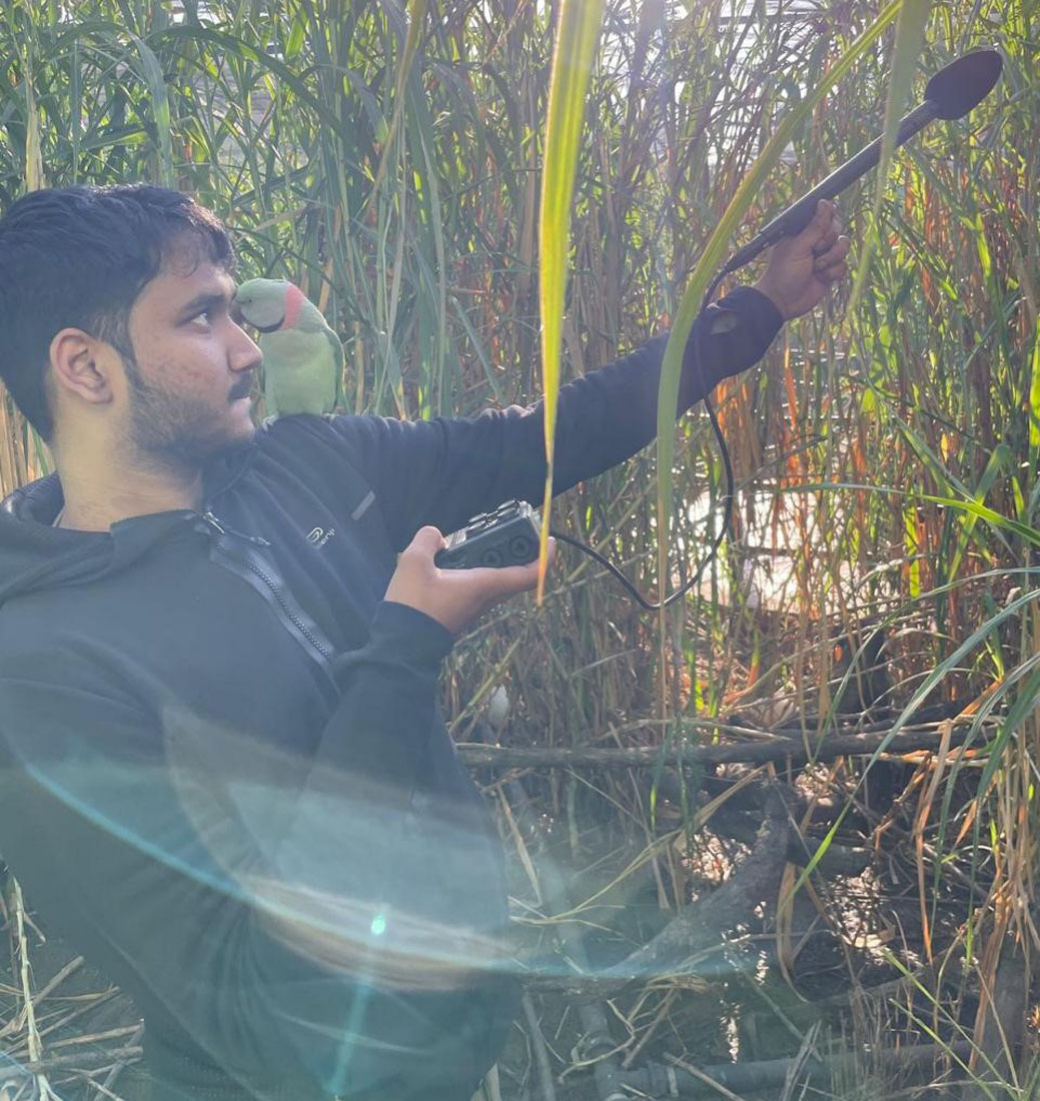


**Nakul Wewhare**

**Describe your scientific journey and your current research focus**

I am currently a 5th-year BS-MS student at IISER Pune. My scientific journey began with studying cognition and vocalizations in free-ranging dogs. This sparked my interest in bioacoustics and led me to work with parrot warble songs, where I became fascinated by their complexity and potential to encode information like a language. Since then, I've worked on various projects analyzing the structure and function of vocal sequences in marmosets and multiple parrot species. Currently for my master's thesis, I am examining how social factors influence the cultural evolution of budgerigar warble song.

I am examining how social factors influence the cultural evolution of budgerigar warble song

**Who or what inspired you to become a scientist?**

I grew up watching National Geographic, Animal Planet, and Discovery TV. It always seemed like the best life to work with animals, but young me thought it meant being either in front of or behind the camera, neither of which I felt suited me. So, I assumed it was just a dream. In high school, I realized that being a scientist is about asking interesting questions and finding answers, which seemed like a good life to me. I decided to join a basic science institute for my undergrad. I was hooked on animal behaviour after reading books by Dr Raghavendra Gadagkar, where I was fascinated by evolutionary explanations of surprising animal behaviours, like lions killing cubs in their own pride and worker bees sacrificing themselves for the queen. Since then, I've worked on various projects in animal behaviour and have become very interested in understanding how animals ‘talk’ to each other and their acoustic communications.

**How would you explain the main finding of your paper?**

Budgerigars sing complex songs and sometimes dance along with their songs. Our study looked at whether different dance moves associate with specific elements of the song, essentially asking if they have a kind of choreography. We found that they do, but each bird's choreography is unique. There appears to be a hierarchy among them: some associations are common across all birds, others are shared by only a few, and some are unique to individuals.

**Figure:**
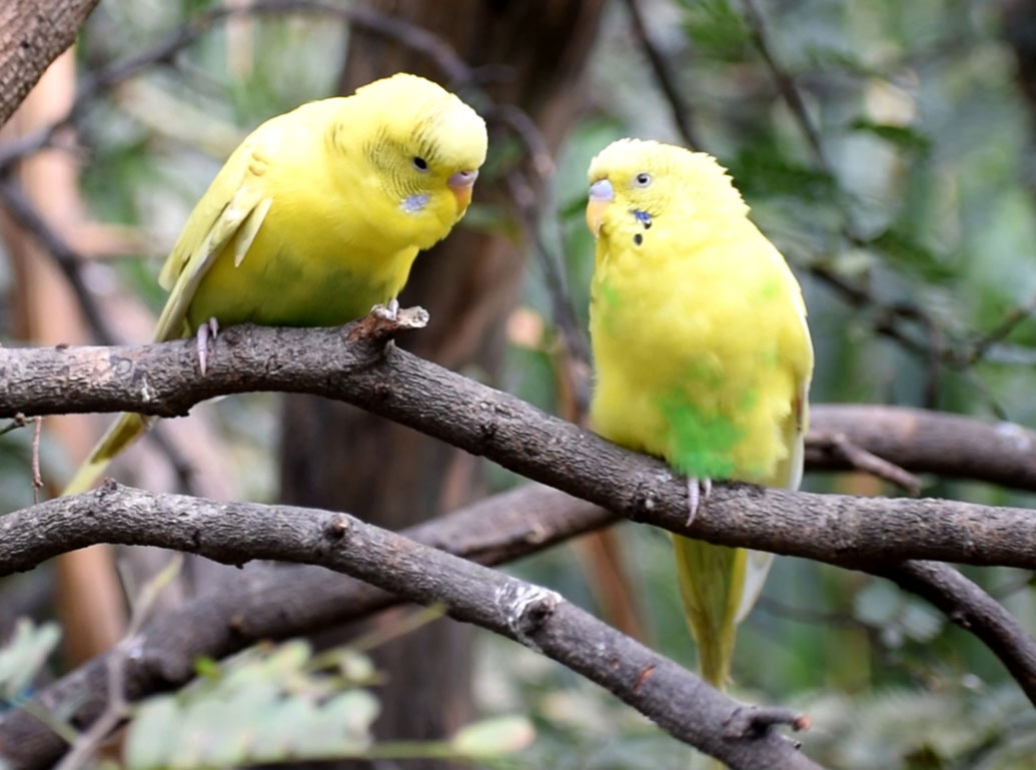
A pair of budgerigars on a branch, with the male on the left in the middle of a courtship display and the female on the right observing it.

**What are the potential implications of this finding for your field of research?**

The finding that budgerigars have hierarchical and individually unique associations between song and body movements suggests that these associations are likely socially learned. This variability, along with group commonalities, could be important features of both group and individual identity. It highlights the need for further research to explore how these associations are shaped by social dynamics and whether they are used for group or individual recognition.

The finding that budgerigars have hierarchical and individually unique associations between song and body movements suggests that these associations are likely socially learned

**Which part of this research project was the most rewarding?**

The most rewarding part of the project was observing the birds in the colony cage, watching them go about their day, and realizing that each parrot had a unique personality. I also had fun giving them funny names based on their personalities.

**What's next for you?**

After finishing my master's thesis, I hope to start as a graduate student in fall 2025 and continue working on complex vocal sequences in animals to better understand how they encode information in these sequences and try to understand how a language evolves.
